# Expanding vaccination competencies to community pharmacists: modelling the organizational and economic impacts of new human papilloma virus (HPV) vaccine pathways in France

**DOI:** 10.1186/s12913-024-11093-x

**Published:** 2024-07-25

**Authors:** Bruno Julia, Claire Foerst, Sarah Akarkoub, Sarah Azzazene, Nathalie Grémaud, Romain Olivier Sénégas, Benoit Mourlat, Carole Mamane, Lionel Bensimon, Gaëlle Farge

**Affiliations:** 1 Community Pharmacist, Lherm, France; 2 IQVIA, Paris, France; 3 MSD, Paris, France

**Keywords:** Human papillomavirus vaccine, Vaccination competencies, Organizational impacts, Economic impact, Pharmacists

## Abstract

**Background:**

The vaccine coverage rate (VCR) for human papillomavirus (HPV) in France is one of the lowest in Europe, well below the target of 80% announced in the French Cancer Plan 2021–2030. The extension of vaccination competencies (prescription and administration) to new health care providers, such as community pharmacists (CPs), was a decisive step by the French Health Authority (HAS) in 2022 to simplify access to vaccination and improve the VCR. This research assessed the economic and organizational impacts (OIs) of the extension of vaccination competencies in France.

**Methods:**

A model was developed in Excel® to compare the current HPV vaccination pathway focused on general practitioners (GPs) to a mix of pathways (new and current) that extends pharmacists’ competencies (prescription and/or injection). The simulated population corresponded to girls and boys targeted by the French recommendations. The model was run from 2023 to 2030. HAS guidelines were used to identify OIs related to these new pathways. Model inputs were collected from national data sources and an acceptability study. The results focused on three OIs (HPV vaccination ability [defined as the number of adolescents who could be vaccinated in each pathway], the VCR projection, and flows of activity between health care professionals]). The economic impact was evaluated from the National Health Insurance (NHI) perspective in 2022.

**Results:**

With a mix of vaccination pathways, including an increasing role of pharmacists, the target of an 80% VCR could be reached in 2030 (versus 2032 with the current pathway) with lower investment than the current situation, resulting in cost savings for the NHI of €212 million. Expanding vaccination competencies will provide pharmacists with additional revenue (an average of €755,000/month for all vaccinating pharmacies) and will free up medical time for GPs (average of 603,000 consultations/year for all GPs).

**Conclusions:**

Expanding vaccination competencies to pharmacists has a positive impact on the entire ecosystem. From a public health perspective, the national VCR target can be achieved and better access to care can be provided, freeing up medical time. From an economic perspective, this approach can provide savings for the NHI and additional revenue for pharmacists.

**Supplementary Information:**

The online version contains supplementary material available at 10.1186/s12913-024-11093-x.

## Background

Most human papillomavirus (HPV) infections self-resolve (90%) [1], but some infections may persist and progress to precancerous lesions and cancers, anogenital warts and recurrent respiratory papillomatosis in men and women [2, 3]. Vaccines can prevent HPV infections. In France, vaccination against HPV infections was first introduced in July 2007 for girls. Since 2021, vaccination against HPV infections has been recommended for girls and boys aged 11 to 14 years (2-dose schedule), with a catch-up vaccination recommended for unvaccinated individuals aged ≤ 19 years (3-dose schedule). The recommendations also target immunocompromised individuals and men who have sex with men (MSM) up to age 26 [4].

The HPV vaccination coverage rate (VCR) in France is one of the lowest in Europe. In 2021, only 37.4% of girls received a full schedule by age 16, and 6.0% of boys received at least one dose [5, 6]. This rate is well below the target of 80% for girls and boys set in the ten-year cancer control strategy 2021–2030 published by the National Cancer Institute (INCa) [7]. An increasing vaccination rate will contribute to eliminating cervical cancer and reducing the incidence and mortality of other HPV-induced cancers [8]. For these reasons, the French government emphasizes prevention as a priority in the fight against cancer and hopes to increase vaccination against HPV [9]. In February 2023, French President Emmanuel Macron announced the launch of a generalized vaccination campaign against HPV in middle schools starting in September 2023 [10].

One of the reasons for this low HPV coverage rate, as highlighted by the HAS, is the complexity of the HPV vaccination pathway [11], which focuses on general practitioners (GPs). Each of the 2 or 3 doses involves a medical visit to obtain a vaccine prescription, delivery of the vaccine at the pharmacy and then another medical visit for its administration. In France, more than 5.4 million people were not registered with regular GPs in 2019 [12], and 30.2% of the French population lived in a medical desert [13].

In this context, the HAS published two recommendations in January and June 2022 to extend vaccination competencies (prescription and administration) to community pharmacists (CPs) for adolescents and adults [14–17]. Alternative pathways involving CPs have existed for the influenza vaccine since 2019 and for COVID-19 since 2021, and these pathways have had a positive impact on the VCR [11]. In this context, the simplification of the vaccination pathway based on the extension of vaccination competencies to additional health care providers, particularly CPs, could be an important support for the adolescent population receiving this vaccination. The international literature shows that enhancing the value of pharmacists’ activity directly contributes to increasing VCR, especially in the United States [18, 19].

With the December 2020 publication by the HAS of its methodological guidelines to assess the organizational impacts (OIs) of health technologies or services [20], there is growing interest in assessing the impacts of new services on health care system organization. From this perspective, it is interesting to model the OI associated with the extension of vaccine competencies to objectively assess the benefits of these new pathways for the ecosystem.

The objective of this research was to assess the economic and organizational impacts associated with the extension of vaccination competencies to CPs in France by comparing new HPV vaccine pathways to the current GP-only pathway.

## Methods

**Fig. 1:**
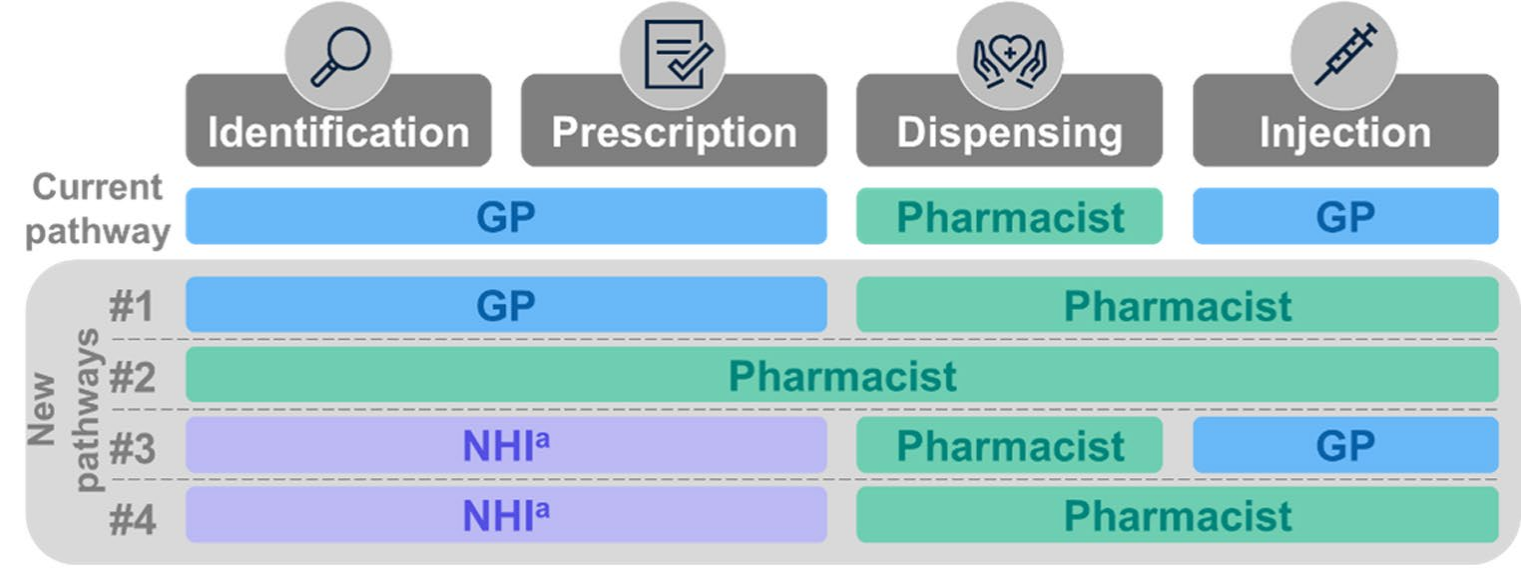
Current and new HPV vaccine pathways. ^a^ Invitations sent by the National Health Insurance (NHI)

The extension of HPV vaccination competencies to CPs was conceptualized in four pathways (Fig. 1). The pathways were defined by identifying all stakeholders who were historically involved (GPs), recently involved (CPs) or potentially involved in HPV vaccination in the next years (National Health Insurance). The pathways were validated with a scientific ad board. In the scenario with a mix of vaccination pathways (called “scenario compared” or “new scenario”), the GP-only current pathway continues to exist alongside the four new complementary pathways. In pathways 3 and 4, parents of eligible adolescents receive an invitation from the National Health Insurance (NHI) that allows them to purchase the vaccine directly from the pharmacy.

### Identification of OI related to the extension of vaccine competencies to new actors

The identification of OIs was based on mapping and the methodology proposed by the HAS [20]. This preliminary qualitative analysis was necessary to identify all the expected OIs associated with the new vaccination pathways and select only those that could be measured using indicators.

### Model structure

The model was developed in Microsoft Excel®, and VBA was utilized to automate calculations and run deterministic and scenario sensitivity analyses. The model compared the GP-only current situation to the mix of different vaccine pathways in which pharmacists’ competencies are extended (prescription and/or administration) in terms of economic and organizational impacts. This model was not based on epidemiological considerations and therefore did not assess the impact of HPV vaccination on the number of cancers prevented.

The model adopted the perspective of the French NHI, as recommended by the HAS [21]. The model was run from 2023 to 2030, consistent with the ten-year cancer control strategy defined by the National Cancer Institute for 2021–2030 (target of 80% VCR) [7].

Evidence from the literature and from an acceptability study conducted by MSD France in 2022 informed the inputs of the model [22]. The objective of this acceptability study was to understand the acceptability according to HCPs and parents of adolescents of the extension of vaccination competencies to community pharmacists for HPV vaccination.

### Estimation of the target population

The eligible population was adolescents targeted by the recommendations (girls and boys aged 11–19 years old). It did not include the MSM population aged 20 to 26 years, for which no data were available. Each year, the eligible population was estimated using the population projections for France for the period 2021–2070 published by the National Institute of Statistics and Economic Studies (INSEE), which is the official French census provider [23].

To account for anti-vaccination movements, the target population was defined as unvaccinated adolescents among the eligible population with confidence in vaccination who were potentially willing to be vaccinated. The probability of not being vaccinated was estimated based on a French study published by Huon JF et al. (2020) that showed that 37.8% of the interviewed parents were in favour of having their children vaccinated against HPV, while 50.4% of parents were undecided. Therefore, it was estimated that 11.8% of parents were against HPV vaccination [24].

### Pathway distribution

In the new scenario, the target population was distributed between the complementary pathways (Table 1). The distribution between the current pathway and the new pathways was based on results from an acceptability study [22] in which parents were asked to rate their level of acceptance of the extension of vaccine competencies (score from 1 = not in favour to 10 = totally in favour). A score of 9–10 was retained for 2023, a score of 8–10 for 2024 and a score of 7–10 for 2025 based on the assumption that more parents would be in favour of extending vaccination competencies over time.

The percentage of adolescents vaccinated via the new pathway will gradually increase, following a linear trend, and is expected to reach 70% (score 6–10) by 2028. It was assumed that the acceptability of these new pathways would stagnate at 70% given a learning curve of 5 years. To split the population between the 4 new pathways between 2023 and 2025, the results of the acceptability study were used with the first position ranked pathway. From 2028 onward, data from a survey conducted by IPSOS in 2021 [25] were used in which 58% of the French population was in favour of vaccination in pharmacies for vaccines such as the HPV vaccine (pathways 1, 2 and 4; an equal distribution was assumed between these 3 pathways). To split the population between the four new pathways in 2026 and 2027, linear evolution (increase or decrease) was applied to each pathway between 2025 and 2028.

**Table 1:** Pathway distribution over the time horizon (2023–2030)

	2023	2024	2025	2026	2027	2028	2029	2030
**Current Pathway/Pathway #0**	75%	56%	39%	36%	34%	30%	30%	30%
**New pathways**	25%	44%	61%	64%	67%	70%	70%	70%
Pathway #1: GP-CP	11%	19%	27%	24%	21%	19%	19%	19%
Pathway #2: CP	3%	5%	7%	12%	15%	20%	20%	20%
Pathway #3: NHI-GP-CP	8%	14%	20%	17%	15%	12%	12%	12%
Pathway #4: NHI-CP	3%	5%	7%	11%	15%	19%	19%	19%

GP: general practitioner; CP: community pharmacist; NHI: National Health Insurance

### Outputs

One economic impact and three organizational impacts were identified as measurable and are described below.

#### HPV vaccination ability

HPV vaccination ability was defined as the estimated number of targeted adolescents who could be vaccinated each year in each pathway. This information was obtained by considering the interactions needed to obtain a full vaccination plan and the number of interactions HCPs can dedicate to HPV vaccination per year. The latter was based on the number of vaccinators (b using the medical demography of each HCP category), their average number of adolescents managed per year, and the share of HPV vaccination among their consultations or activity. For GPs, an interaction was defined as a prescription consultation or an injection consultation. It was estimated that 103 interactions per GP and per year could be dedicated to HPV vaccination [26–30]. For pharmacists, an interaction was defined as a prescription and injection or an injection without prescription. It was estimated that 551 interactions per pharmacy and per year could be dedicated to HPV vaccination [26, 31, 32].

The evolution of medical demography is updated each year based on French National Agency for Economic Statistics (DREES) data for GPs and data from the Ordre National des Pharmaciens (*National Pharmacists Association*) for pharmacies [27, 31]. We focused on 94% of French pharmacies, corresponding to the 18,689 pharmacies allowed to carry out vaccination (Order of April 23, 2019) [31].

#### Vaccination coverage rate projection

The VCR projection was estimated using the Santé Publique France (SPF) calculation method [5]. The cumulative number of vaccinated adolescents in year N was estimated based on the number of previously vaccinated adolescents (in year N-1) plus the targeted adolescents who can be vaccinated per year and who effectively are vaccinated, which depends on their intention to be vaccinated.

In the current study, girls’ intention was estimated based on available historical VCRs and projected from 2022 to 2030 using linear regression. For boys, intention was estimated based on the 2021 VCR. It was assumed that in 2030, boys’ vaccine intention would be equal to girls’ vaccine intention based on the US experience; that is, it would take 6 years for boys to reach girls’ VCR. Thus, boys’ vaccine intentions follow exponential growth. The intention reached 40% in 2030 for girls and boys.

In 2023, the intention to be vaccinated through the new HPV vaccination pathways was derived from an acceptability study conducted by MSD France in 2022 (the question asked was “If the pathway you prefer were implemented, would you vaccinate your adolescents aged 11–19 years against HPV?”). It was assumed that due to the expected benefits associated with the extension of vaccination competencies, the intention in 2030 would be slightly greater than the current situation and would reach 50% (consistent with values observed for the influenza vaccine in recent years). Between 2023 and 2030, the intention was estimated with a linear regression.

#### Flows of activity between HCPs

The revaluation of flows of activity between HCPs aims to calculate the number of interactions/consultations related to HPV vaccination gained or released by each HCP with the mix of new pathways compared to the current situation.

The average duration of an interaction related to HPV vaccination for GPs was used to estimate the number of consultations by GPs in each pathway.

The total number of interactions related to HPV vaccination in the new scenario and in the GP-only current pathway was converted into a financial flow depending on the revenue associated with each interaction per HCP.

#### Economic impact

The economic impact aims to calculate the difference in terms of costs between the new scenario and the GP-only current pathway. The total investment cost to reach an 80% VCR over the period from 2023 to the year of achievement of the 80% VCR in the scenario with the mix of vaccination pathways and in the GP-only current pathway was calculated as well as the additional revenue generated for pharmacies based on the flows of activity transferred.

Consistent with the HAS recommendations [21], from the NHI perspective only direct costs, including the cost of Gardasil 9® [33], prescription and/or administration tariffs for pharmacists [34], GP consultation and the cost of NHI invitation, were considered and estimated. The cost of NHI invitation was estimated at 0.026€ based on the cost of sending one text message for a campaign for more than 1 million people [31].

### Sensitivity analyses

The robustness of the model was assessed through sensitivity and scenario analyses.

#### Univariate deterministic sensitivity analysis

(DSA) was performed on the 30 parameters listed in Appendix 2. DSA was conducted by varying one parameter at a time on an upper bound and a lower bound. These bounds were the 95% confidence interval when available or an arbitrary value set at ± 10%. DSA was performed for two outcomes: the difference in the total number of vaccinated patients (DSA #1) and the difference in the average annual economic impact for 2023–2030 (DSA #2).

#### Scenario analyses

Were used to assess the impact of alternative structural assumptions on the results. Six scenario analyses were conducted, including a higher target of vaccine intention in 2030, a higher VCR for boys in 2021, a different distribution of pathways in the new scenario, and a linear increase in boys’ vaccine intention.

### Verification and validation of the modelling

At each stage of the model’s development, verification was undertaken in both the formulas and the visual basic for applications (VBA) macros. Verifications were performed by two of the authors involved in the model’s development and an external reviewer.

## Results

### HPV vaccination ability

Considering that there were 93,584 GPs in France in 2022, each of whom was able to perform 103 theoretical HPV vaccinations per year, the maximum number of interactions that could be dedicated by GPs to HPV vaccination in the current situation was estimated to be 9.6 million per year [27]. With an average of 20.1 million possible interactions per year considering the mix of pathways, the complementary action of GPs and pharmacists in the scenario with the mix of vaccination pathways allows more than 10.5 million more possible interactions per year for HPV vaccination. This represents a 110% increase in appointment opportunities related to HPV vaccination (prescription and injection) compared to the current scenario.

In the French situation, before the new pathways were implemented, it was estimated that 2.7 million adolescents could be vaccinated per year if 9.6 million interactions were used. New vaccination pathways are associated with a total of 7.3 million adolescents who could be vaccinated per year, corresponding to 4.6 million more adolescents than the “GP-only pathway” (+ 167%).

### Projection of the vaccination coverage rate

If the current situation were to persist, the 80% target VCR would be reached for all adolescents (girls and boys) in 2032 versus 2030 (objective set by INCa) with the extension of vaccination competencies.

### Flows of activity between HCPs

In the “GP-only pathway”, GPs dedicate, on average, 2,783,300 consultations to vaccinate eligible adolescents against HPV per year over the period 2023–2030. With the complementary action of pharmacists, 602,868 of these consultations (22%) could be dedicated each year to medical activities other than HPV vaccination. This is of particular interest in areas with low medical density.

The difference between 2023 and 2030 in HPV-related interactions highlights a gain in activity for pharmacies. In 2023, the average numbers of injections (a mixture of pathways 1 and 4) and prescriptions + injections (pathway 2) were 2.00 and 0.6, respectively, per month per pharmacy, while they were 4.0 and 2.1, respectively, in 2030. Therefore, an average of 6 HPV-related interactions per month are dedicated per pharmacy over the period 2023–2030 (4 injections and 2 prescriptions + injections).

### Economic impact

The investment cost to reach an 80% VCR with the mix of vaccination pathways (2023–2030) is approximately €1.9 billion versus €2.1 billion with the current situation (2023–2032). Expanding vaccination competencies will result in cost savings of €212 million to reach the target of an 80% VCR compared to the current situation.

#### Additional revenue generated for pharmacists for HPV vaccination

Expanding vaccination competencies could generate an average additional revenue of €755,000/month for all vaccinating pharmacies. This overall gain represents additional revenue of €580/year per pharmacy.

### Sensitivity analysis

**Fig. 2:**
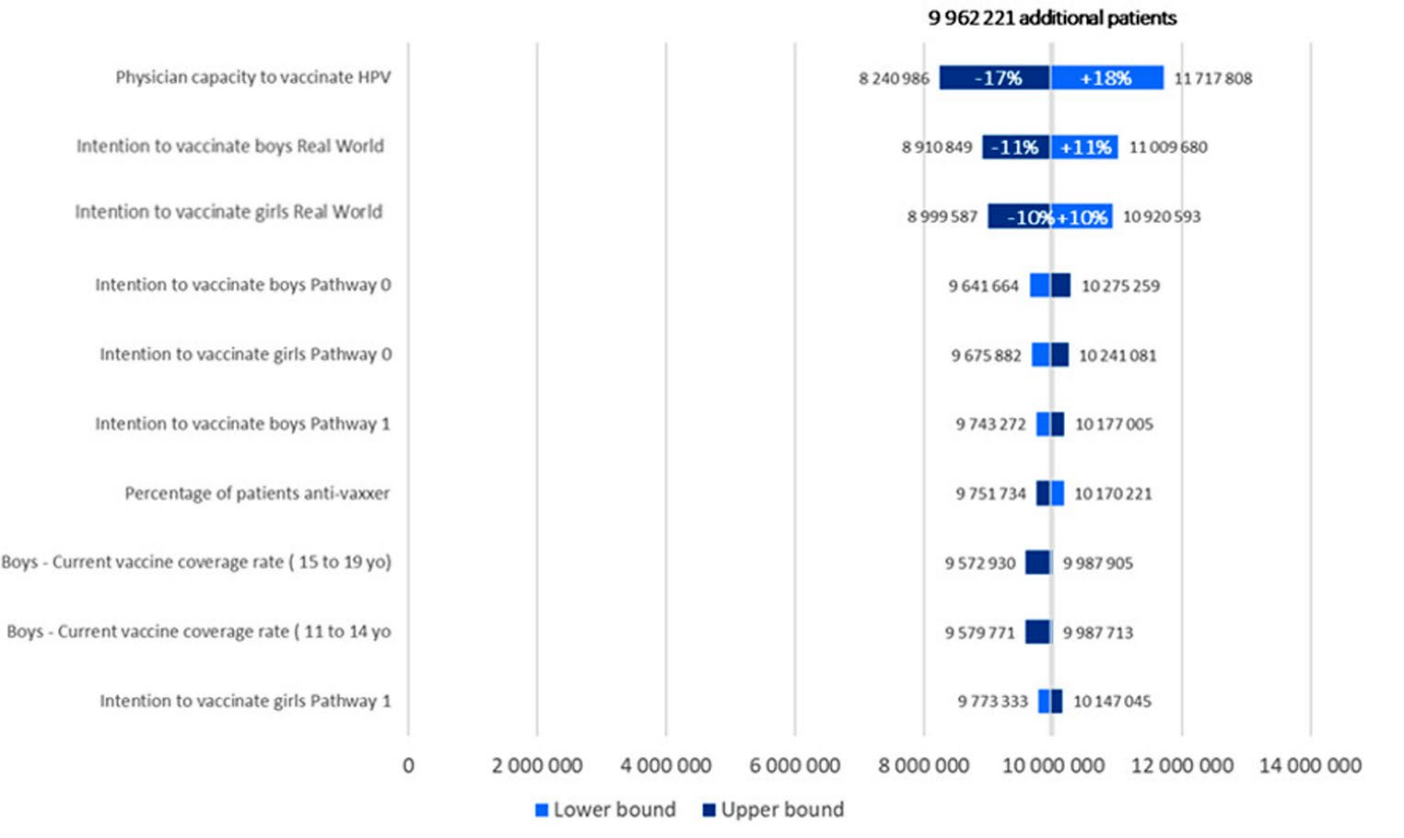
Tornado diagram - variation in the number of vaccinated adolescents (2023–2030)

**Fig. 3:**
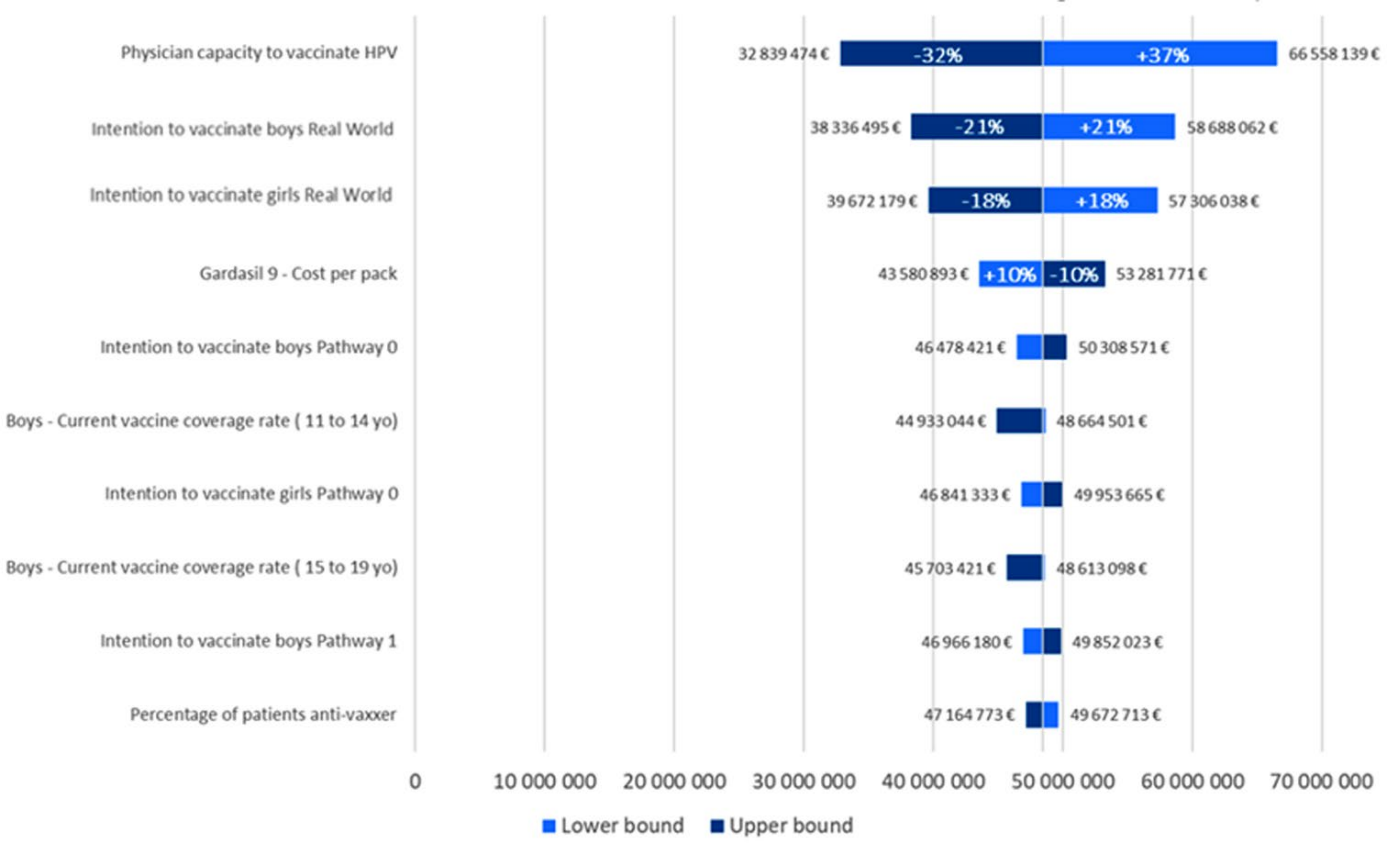
Tornado diagram - average annual economic impact

The DSA results showed that uncertainty was mainly driven by physicians’ ability to vaccinate against HPV. Additionally, in the GP-only current pathway, boys’ and girls’ willingness to be vaccinated was a driver (Figs. 2 and 3).

The results from the scenario analyses are presented in Appendix 1. Comparable results to the base-case analysis were found for both the VCR and economic impact. Nevertheless, an 80% VCR could be reached more rapidly with assumptions of a higher target of vaccine intention in 2030 and a higher share of vaccination among GP consultation. A better economy for the NHI could be observed with a higher VCR of boys in 2021, with a different distribution of pathways in the new scenario, with a higher number of GP interactions for the current situation/pathway 0 and with linear growth of boys’ vaccine intention.

## Discussion

The model enabled a comparative analysis of the economic consequences and organizational impacts of simplifying the HPV vaccination pathway with the extension of vaccination competencies (prescription and administration) to community pharmacists. To our knowledge, this research is the first to explore the measurement of OIs related to new vaccination pathways.

The increase in the HPV VCR enabled by the new vaccination pathways after the expansion of vaccination skills is conditional on the implementation of information campaigns. Training for pharmacists and communication campaigns for the general public must be implemented for this purpose [35, 36]. Recent announcements by French President Emmanuel Macron about school-based HPV vaccination [10] could positively impact the model outcomes and would certainly contribute to faster achievement of the 80% VCR in 2030. However, the model was developed prior to the recent official announcement of an upcoming school-based vaccination campaign and therefore did not consider this new mode of vaccination.

Social environments play a determining role in shaping the acceptance of vaccination among populations. Indeed, it has been acknowledged that the acceptance of HPV vaccination among targeted populations differs according to cultural and socioeconomic factors. Several variables have a significant impact on the acceptance of vaccination, including religious convictions, educational attainment, residential location (urban or rural), socioeconomic status, the degree of interest in alternative medicine and anti-vaccine movements, and the age of the patient [37, 38]. Several factors at the departmental level were also found to be significantly associated with vaccination rates. Specifically, unemployment, the proportion of immigrants and foreigners, and limited coverage by CMU health insurance were negatively correlated with vaccination rates [39]. Therefore, minority groups are more inclined not to be vaccinated. By understanding the complex interplay among these factors, we can develop more effective strategies to promote vaccine uptake and improve global health outcomes.

The current momentum to enhance the value of pharmacist activities is strongly based on the extension of their competencies and on their accessibility as community HCPs. Vaccination is one of the recent examples of this dynamic. This phenomenon is part of a global movement to strengthen the role of pharmacists, which has been underway for many years, including the re-evaluation of the act of dispensing and the development of new remuneration schemes as well as the involvement of pharmacists in patient pathways (care management) [40, 41].

Several structural limitations of the model were identified. The developed model is a simple static model and does not consider the epidemiological impact (cancers and HPV-related diseases avoided). The objective of this model is to identify the measurable economic and organizational impacts of vaccine competency expansion with a focus on adolescents eligible for HPV vaccination. Only OIs that could be measured using indicators were included in the model. However, the OI related to the extension of vaccine competencies goes beyond these measurable OIs in the model and includes quicker access to vaccines (reduced delays in access to vaccination appointments) and impacts related to HCP coordination. Another limitation is that data from the acceptability study (intention to vaccinate, pathway distribution, share of HPV vaccination among HCP activity) are declarative data from a study conducted with participants who were asked to project themselves into new hypothetical scenarios. Therefore, the impact of these policy changes will be updated with real-world data, especially annual updates of the VCR, as well as the real-life adoption of these new vaccination pathways (the distribution of eligible adolescents according to pathways).

## Conclusions

The results of the base-case analysis showed that with the mix of vaccination pathways, if the GP-only current pathway continues to exist alongside the new complementary pathways involving pharmacists, the target of an 80% VCR for girls and boys in 2030 could be reached in accordance with the objective set by the INCa, which would contribute to eliminating HPV-related cancers. In addition to this positive public health impact, this new organization of care would allow GPs to dedicate 603,000 consultations per year to other medical activities and would address the lack of available GPs, especially in areas with low medical density. This release of medical time for GPs would be made possible by the complementary action of pharmacists, which would generate additional revenue of approximately €755,000 per month for all vaccinating pharmacies. The extension of vaccination competencies would permit the NHI to save €212 million while reaching the 80% VCR target. These savings are necessary and sought by the authorities to ensure the sustainability of the French health system.

Deterministic and scenario-based sensitivity analyses were conducted to assess parameter and structural uncertainty. The results were robust across sensitivity analyses.

This model showed that new vaccination pathways can generate savings. In the area of prevention, costs occur from a short-term perspective, while health outcomes occur from a medium- to long-term perspective. The model does not take into account the effects on cancers and pathologies due to the prevention of HPV infections.

This model highlights the economic and organizational benefits of a recommendation published by the HAS regarding the extension of vaccination competencies.

To conclude, expanding vaccine competencies (prescription and administration) to community pharmacists has a positive impact on the entire ecosystem, from a public health perspective. It can contribute to the achievement of the national VCR target in 2030 to eliminate HPV-related cancers, better access to care (freeing up medical time) and, from an economic perspective, savings for the NHI and additional remuneration for pharmacists.

### Electronic supplementary material

Below is the link to the electronic supplementary material.

Supplementary Material 1

Supplementary Material 2

## Data Availability

All data generated or analysed during this study are included in this published article.

## Abbreviations

CP: Community pharmacist
CMU: Supplementary health insurance
DREES: French National Agency for Economic Statistics
DSA: Deterministic sensitivity analysis
GP: General practitioner
HAS: French Health Authority
HCP: Health care professionals
HPV: Human papilloma virus
INCa: National Cancer Institute
INSEE: French National Institute of Statistics and Economic Studies
MSM: Men who have sex with men
NHI: National Health Insurance
OI: Organizational impact
SPF: Santé Publique France
VBA: Visual Basic for Applications
VCR: Vaccine coverage rate
